# Orthodontic Treatment of Bilateral Transposition of Maxillary Canines and Lateral Incisors

**DOI:** 10.1155/2022/8094008

**Published:** 2022-01-04

**Authors:** Mandla Dominic Nyakale

**Affiliations:** Department of Orthodontics, Faculty of Dentistry, University of the Western Cape, Cape Town, South Africa

## Abstract

Dental transposition is a severe disturbance of tooth position and its eruptive sequence. It may affect any tooth, and it may occur at any location within the dental arch, although some teeth may be more frequently affected than others. There are several types of dental transposition, and their classification depends on the type of teeth involved. The aetiology of transposition is not very clear and has always been the subject of controversies, and it is still not completely understood. The prevalence of dental transposition and the factors related to this dental anomaly have been well documented in the literature. When treating dental transposition, many factors which may affect the treatment results must be considered, such as aesthetics, occlusion, treatment duration, patient comfort, patient cooperation, and periodontal support. Various treatment options are currently available for the treatment of dental transposition. Successful treatment outcomes depend on the patient's age, concern about facial and dental aesthetics, motivation of the patient, functional requirements, type of malocclusion, and the position of the root apices. The present case report is aimed at outlining the orthodontic treatment of a young adult patient diagnosed with bilateral maxillary canine-lateral incisor transposition.

## 1. Introduction

Dental transposition was first described by Harris [1] as the positional interchange of two adjacent teeth within the same quadrant. Dental transposition may be considered to be complete when both the crowns and the roots of the involved teeth are completely interchanged and as incomplete when only the crowns of the involved teeth are interchanged, while their roots remain in their normal positions [2]. Dental transposition can adversely affect both the aesthetic and functional aspects of the dentition [3], and thus, it is important to understand the occurrence of this dental anomaly in order to manage it timeously and efficiently [4]. A multidisciplinary approach may be needed for the successful treatment of this dental anomaly in order to achieve long-term aesthetic and functional outcomes [3, 5–7]. The prevalence of dental transposition has been shown to vary in different populations. The highest prevalence of dental transposition has been reported in the Chinese population at 0.81% [8], followed by Africa estimated at 0.51% [9, 10], the United Kingdom estimated at 0.38% [11], and the European population with the least prevalence estimated at 0.26% [12]. Numerous studies have shown that dental transposition occurs more frequently in females than in males [3, 13–18] although other authors have reported a higher frequency in males [19–22]. The maxillary arch has been shown to be affected more frequently by dental transposition than the mandibular arch [22–24]. The tooth most frequently affected by dental transposition is the maxillary canine [14] with a prevalence of 0.14-0.51% [25, 26], and unilateral canine transpositions are more prevalent on the left than the right side [27]. Maxillary canine-first premolar transposition has been reported to be the most frequent, with a prevalence rate of approximately 0.13% [14, 28] in the general population [29]. Peck and Peck [16] classified dental transposition into five categories according to the type of teeth involved and also in the order of decreasing frequency as follows:

1. *Class I*. Canine-first premolar transposition
2. *Class II*. Canine-lateral incisor transposition
3. *Class III*. Canine-first molar transposition
4. *Class IV*. Lateral incisor-central incisor transposition
5. *Class V*. Canine-central incisor transposition

Several factors have been implicated in the aetiology of dental transposition, and these include genetic factors [2, 30], interchange in the position of the developing dental lamina during odontogenesis [14], trauma [13], mechanical interferences [16], and early loss of primary teeth [3, 15, 17, 18, 20, 21, 31]. Dental transposition may also occur with other dental anomalies such as tooth agenesis [15, 32], peg shaped lateral incisors [3], and retained primary teeth [33]. Diagnosis is usually made clinically; however, radiographs may be needed to confirm the severity of this dental anomaly. Early treatment is suggested for this condition [34]; however, this may require complex and lengthy treatment methods [35].

Presence of dental transposition can also be a very useful tool in the field of forensic odontology [36]. Over the years, dental transposition has been used successfully to identify unknown individuals, deceased victims, suspects of a crime, and also the victims of a mass disaster. Due to its extremely rare occurrence, dental transposition can be a very useful marker on the dental records which can be used to identify individuals, particularly when other comparative methods of identification are neither available nor adequate to establish the identity of the individual in question [36].

The objectives of treatment are to establish good static and functional occlusion and also to provide pleasing facial aesthetics while maintaining temporomandibular joint and periodontal health [37]. The treatment of dental transposition frequently requires a multidisciplinary approach in order to achieve long-term aesthetic and functional results [3, 4, 6, 7, 38]. When treating dental transposition, many factors that may affect the treatment results must be considered, such as the patient's age, dentofacial aesthetics, functional occlusal requirements, treatment duration, patient motivation and cooperation, periodontal support and type, and severity of malocclusion. Age is the single most important factor beyond all the other factors, which is directly correlated with the tissue regeneration. Available treatment options include alignment of the transposed teeth in their transposed positions [19, 39], extraction of one of the transposed teeth, or complete correction of the transposed teeth to their normal positions [40]. Many authors believe that the ideal treatment approach is to completely correct the transposed teeth to their normal positions; however, this may not be possible in many clinical situations. Risk to the teeth and adjacent tissues and treatment duration must be assessed and discussed with the patient prior to orthodontic treatment. The following case report is aimed at presenting the orthodontic treatment of a patient diagnosed with bilateral maxillary canine-lateral incisor transposition.

## 2. Case Presentation

**Figure 1:**
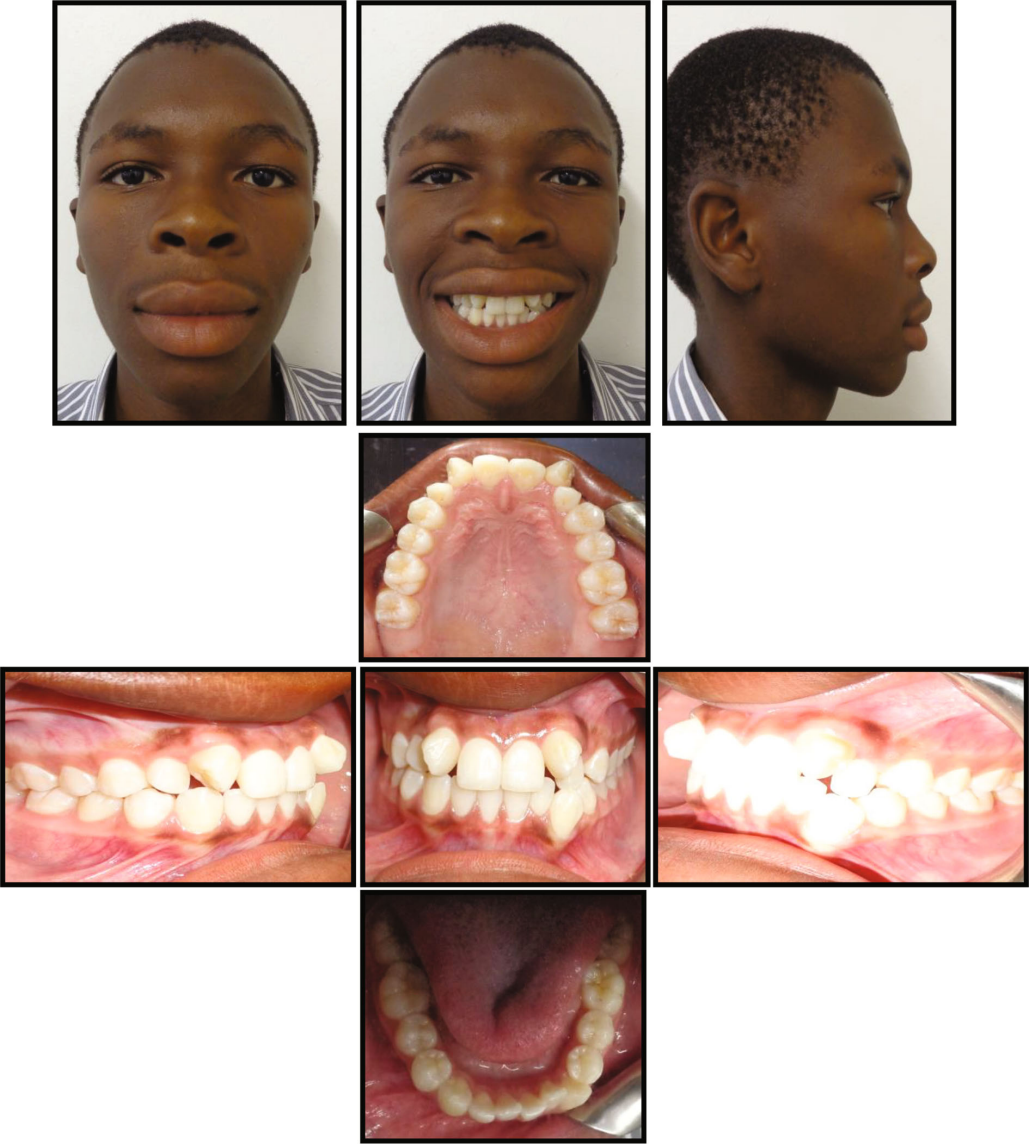
Pretreatment photograph.

**Figure 2:**
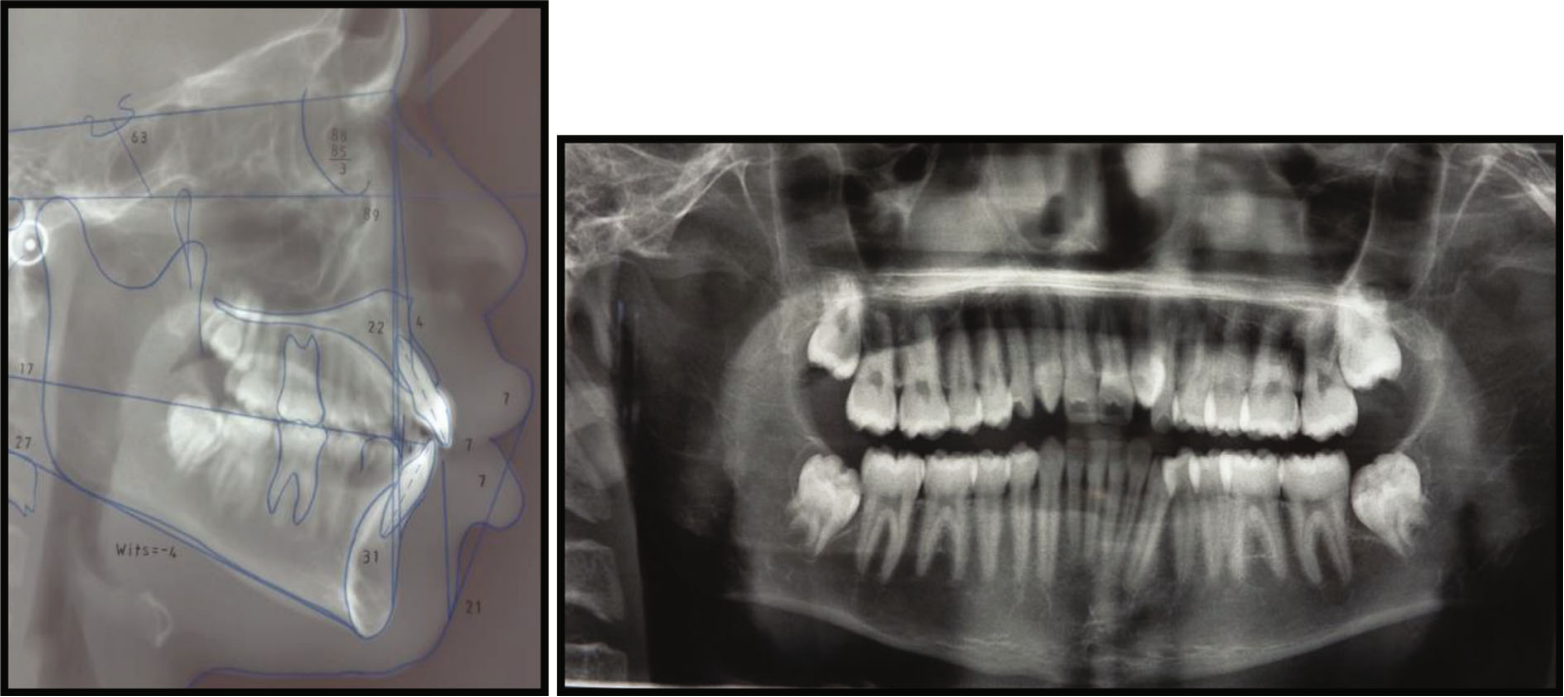
Pretreatment radiographs.

An 18-year old male patient was referred to the orthodontic clinic at Pelonomi Tertiary hospital in Bloemfontein (Free State Province, South Africa) with the chief complaint of crowded anterior teeth and poor aesthetics. Ethical approval to publish this case report was obtained from the Health Sciences Research Ethics Committee of the University of the Free State (Ethics Reference number: UFS-HSD2019/0697/0110, Appendix A). The patient was in good general health, and the medical and dental history indicated no contraindications to orthodontic treatment. The patient had no history of trauma to the head and neck area. Extraoral examination revealed a normal face type, a convex facial profile with a symmetrical face. Intraoral examination showed the permanent dentition stage, good oral hygiene with no deleterious oral habits. The teeth were generally healthy with no carious lesions. The periodontal and gingival tissues were generally healthy with no evidence of bleeding or deep probing depths. Analysis of the occlusion revealed angle class I malocclusion with significant crowding of the maxillary and mandibular anterior teeth which were also protrusive. Bilateral transposition of maxillary canines and lateral incisors were also observed (Figure 1). Panoramic radiographic examination showed the teeth in a permanent dentition stage with bilateral complete transposition of maxillary canines and lateral incisors. The third molars were present in both jaws, and the mandibular third molars were developing in the ramus (Figure 2). No other pathologies were detected on the radiographs. Tracing and analysis of the lateral cephalogram showed a mild class III skeletal pattern with a horizontal growth pattern (Table 1).

## 3. Treatment Objectives

There were several potential treatment options available, ranging from extraction treatment, surgical repositioning of the transposed teeth, and alignment of the transposed teeth in their transposed positions. When planning to align the transposed maxillary canines and lateral incisors in their transposed positions, there are generally two problems to consider. The first problem is whether the lateral incisor will be able to function as a canine [7] and, the second is, whether we would be able to disguise the canine as a lateral incisor [41, 42]. This treatment option was not a preferred choice as our patient had significant crowding of the anterior teeth which required extraction of the first premolars to create space. The maxillary lateral incisors are also less favourable for canine guidance during functional occlusion because of their thin and short roots. Hence, conversion into group function may be beneficial for nonextraction cases [41, 42]. Similarly, camouflage of the maxillary canines will often require grinding of the tips, together with the addition of composite resin or porcelain veneers to improve the aesthetic appearance [41, 42]. The maxillary canine also has a broader and higher gingival contour when compared with the lateral incisor [7], and this option would have compromised the aesthetic appearance. It was mainly for these reasons that it was decided to correct the transposed teeth to their normal positions in the present case. The treatment objectives were to correct the transposed teeth to their normal positions, level and align the dental arches, and establish pleasing dentofacial aesthetics and good functional occlusion. The treatment plan included extraction of maxillary and mandibular first premolars in order to gain the space required to align and upright the crowded and protrusive anterior teeth.

## 4. Treatment Progress

**Figure 3:**
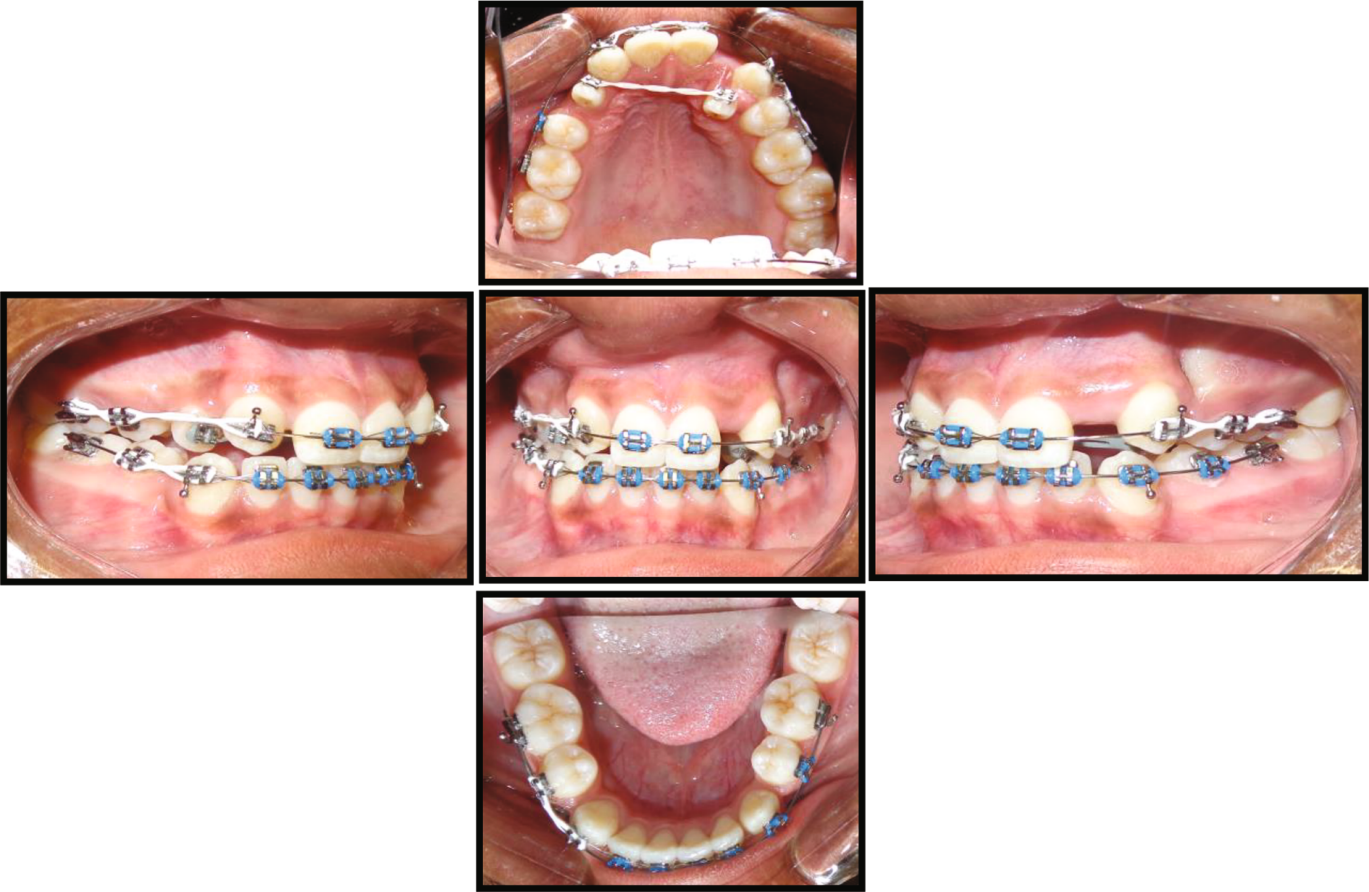
Intermediate intraoral photographs.

Banding and bonding of the teeth were done with the standard preadjusted 0.018 × 0.025-inch slot Roth prescription edgewise appliances. Teeth were levelled and aligned with 0.012-inch, 0.014-inch, and 0.016-inch superelastic nickel titanium archwires, respectively. This was followed with the retraction of maxillary and mandibular canines using elastic chains on 0.016-inch stainless steel archwires. Retraction of the canines was done simultaneously while maxillary lateral incisors were reciprocally displaced towards the palate using elastic chains (Figure 3). The lateral incisors were moved palatally in order to clear the way for the distal movement of the transposed canines. This was also done to prevent bony loss at the cortical plate of the canines and to avoid root proximity between the canine and the lateral incisor thus avoiding root resorption and periodontal breakdown. Levelling and aligning archwires were placed again to finalize alignment of all the teeth including the maxillary lateral incisors. Torque control was initiated with 0.016 × 0.022-inch superelastic nickel titanium, followed by 0.017 × 0.025-inch beta titanium archwires, respectively. Final detailing of the occlusion was done on 0.018 × 0.025-inch stainless steel archwires using finishing elastics. Final rectangular stainless steel wires were left in place for an additional 8 weeks to establish proper root torque. At the end of treatment, the orthodontic appliances were removed, and maxillary Hawley and mandibular fixed 3-3 retainers were placed.

## 5. Treatment Results

**Figure 4:**
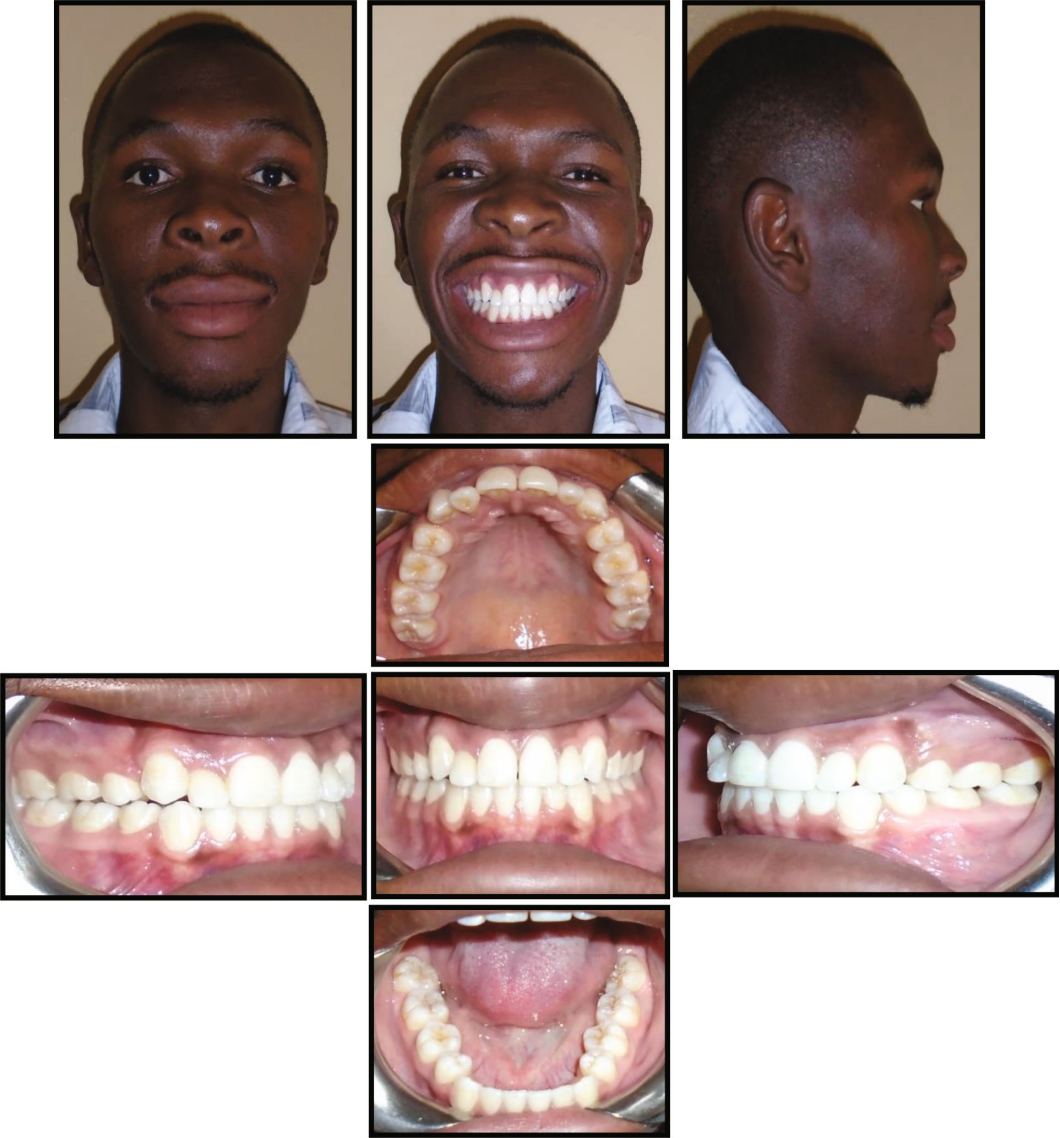
Posttreatment photographs.

**Figure 5:**
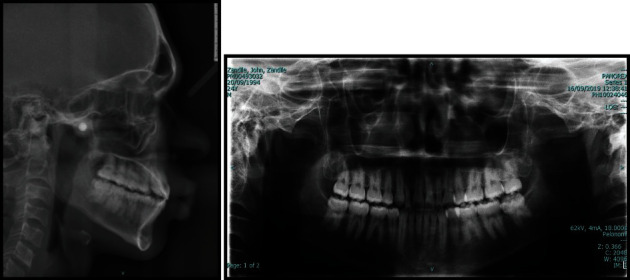
Posttreatment radiographs.

**Figure 6:**
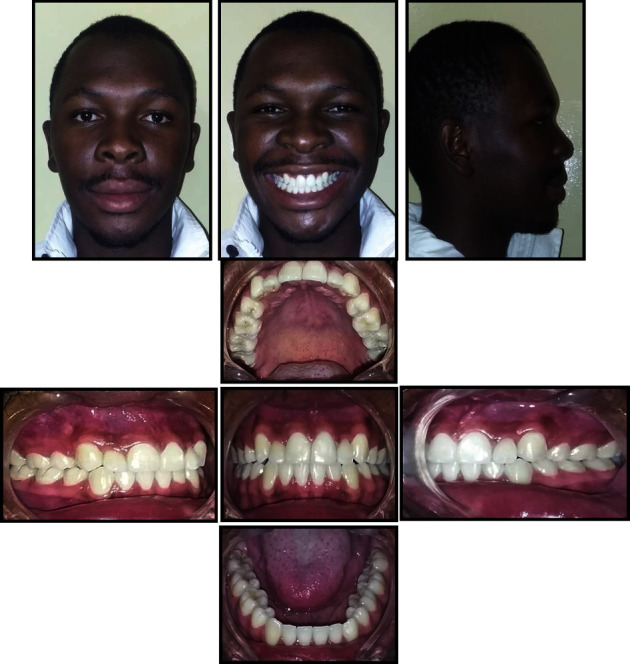
Postretention photographs 14-month postretention.

Treatment lasted for 24 months and at the end of orthodontic treatment, the teeth were well aligned, and canines and molars were in a class I relationship, with normal overjet and overbite. The maxillary and mandibular midlines were corresponding, and this improved the smile appearance. The gingival contour on the palatal aspect of the maxillary right lateral incisor was very high after orthodontic treatment (Figure 4). The patient was referred to the periodontist for the placement of a connective tissue graft to the affected area, but later he declined to have this treatment done. A posttreatment panoramic radiograph showed the optimal positioning of the maxillary and mandibular teeth with proper root parallelism with no signs of alveolar bone or root resorption (Figure 5). Periapical radiographs are more preferred to evaluate the presence of external apical root resorption [43], but unfortunately, these radiographs were not available in the hospital. A posttreatment lateral cephalometric radiograph showed a balanced facial profile with ideal incisor inclinations and no significant changes in the skeletal measurements (Figure 5/Table 1). No evidence of relapse or significant changes was observed 14 months after the retention period (Figure 6/Table 1), and the patient was happy with the treatment results.

## 6. Discussion

This is a case report of an 18-year old male patient who presented to the orthodontic clinic at Pelonomi Tertiary Hospital in Bloemfontein, South Africa. Our patient's age falls within the age range of the majority of patients who seek orthodontic treatment at Pelonomi Tertiary Hospital. Treatment results of this case will assist with treatment planning of future cases with a similar diagnosis. Although it has been reported in the literature that preventive and interceptive treatment of transposed teeth is best instituted early in the mixed dentition stage during the development of the roots of the teeth [3], the results of this case report will give us some insight into the management of transposition which may be diagnosed later in life. Dental transposition has also been reported to occur more frequently in female subjects than male subjects [3, 13–18]. Our patient was male, and this was in keeping with findings by other authors [19–22]. Our case was bilateral transposition of the maxillary canines and lateral incisors, and this has been reported as the second most frequent dental transposition to occur in the human dentition [16], with both dental and aesthetic complications [22]. As discussed earlier, dental transposition may be classified as complete or incomplete depending on the involvement of the crowns and roots [17]. Our case was a complete bilateral transposition of maxillary lateral incisors and canines. Since the maxillary canines are keystones in the dental arch, both for aesthetics and normal occlusal function [41, 42], it was decided to correct the transposed teeth to their normal positions in the present case. This case presented with interesting treatment options, and a simple approach would have been to align the teeth in their transposed positions. Unfortunately, the ideal aesthetic and occlusal considerations suggested that the teeth should be placed in their natural positions. Therefore, alignment of the transposed teeth to their natural positions in the dental arch was a preferred treatment option. It was then decided to extract the four first premolars to create the space needed to align the teeth and also to gain room to correct the transposed teeth. The treatment approach was planned in line with the patient's age, concern about dentofacial aesthetics, and also functional occlusal requirements. Preventive as well as early interceptive treatment in the mixed dentition would have made it easier to correct this anomaly [40], because the bone is usually less dense, and the roots may still be in their developing stages. Dental transposition is one of the rare developmental anomalies that can affect dentofacial aesthetics and occlusal function [2, 11, 37]. To the best of our knowledge, such a rare case of bilateral transposition of maxillary canines and lateral incisors has never been reported in the literature. The outcome of treatment of this case report will give us some insight with regards to the treatment options available to us regarding this dental anomaly. Complete dental transposition, although complicated, can be treated successfully by applying appropriate orthodontic mechanics [40]. The patient's needs should be assessed carefully as the anterior teeth relate directly to the psychological wellbeing of the patient [44]. Treatment of this case also presented with a few challenges, and the biggest challenge was poor buccal root torque control on the maxillary lateral incisors and also the recession on the palatal gingiva on the maxillary right lateral incisor. Proper torque control on the teeth requires a large moment to force ratio of at least 12 : 1 [45], and this can be achieved by placing a full thickness wire for a longer period of time, but unfortunately, the patient wanted to end the treatment as he was happy with the achieved results despite having been advised otherwise. The patient was also referred to the periodontist for the placement of a connective tissue graft, but he later declined to undertake this treatment.

## 7. Conclusion

Although many authors would suggest that repositioning the completely transposed teeth to their normal positions may be a complex and time-consuming process, it was decided in this case report to attempt to reposition the completely transposed maxillary canines and lateral incisors to their normal positions. In the present case, good functional occlusion and pleasing dentofacial aesthetics were achieved. This case report suggests that ideal treatment results can be obtained in difficult cases by meticulous treatment planning and proper mechanics in the shortest time and with the minimum tissue damage possible.

## Figures and Tables

**Table 1:** Lateral cephalometric readings.

Measurement	Norm	Before treatment	After treatment	14-month follow-up
Age		18 years 02 months	20 years 10 months	22 years 01 month
Skeletal pattern				
SNA (°)	87°	88°	87°	88°
SNB (°)	82°	85°	84°	84°
ANB (°)	5°	3°	3°	4°
Face plane angle	87°	89°	88°	90°
Convexity (NA-Apo) (mm)	4 mm	4 mm	3 mm	4 mm
Wits (mm)	-1-2 mm	-4 mm	-3 mm	-3 mm
Occipital plane to SN (°)	7°	7°	7°	7°
S.n. GoGn (°)	32-34°	27°	29°	28°
*y*-Axis (SGn.SN) (°)	66-68°	63°	65°	64°
Incisor relations				
U1-NA (°)	22°	22°	21°	23°
U1-NA (mm)	7 mm	7 mm	5 mm	6 mm
U1-NB (°)	38°	31°	29°	32°
L1-NB (mm)	10 mm	7 mm	6 mm	8 mm
APo (mm)	8 mm	7 mm	6 mm	8 mm
Soft tissues				
Holdaway angle (°)	20°	21°	20°	20°

## References

[1] Harris C. A. (1849). *A Dictionary of Dental Sciences, Bibliography and Medical Terminology*.

[2] Chattopadhyay A., Srinivas K. (1996). Transposition of teeth and genetic etiology. *Angle Orthodontist*.

[3] Shapira Y., Kuftinec M. M. (2001). Maxillary tooth transpositions: characteristic features and accompanying dental anomalies. *American Journal of Orthodontics and Dentofacial Orthopedics*.

[4] Chaushu S., Becker A., Zalkind M. (2001). Prosthetic considerations in the restoration of orthodontically treated maxillary lateral incisors to replace missing central incisors: a clinical report. *Journal of Prosthetic Dentistry*.

[5] Sabri R., Zaher A., Kassem H. (2008). Tooth transposition: a review and clinical considerations for treatment. *Journal of the World Federation of Orthodontics*.

[6] Beznos C. (1996). An alternative approach to replacement of a congenitally missing maxillary central incisor: a case report. *Quintessence International*.

[7] Kokich V. G., Nappen D. L., Shapiro P. A. (1984). Gingival contour and clinical crown length: their effect on the esthetic appearance of maxillary anterior teeth. *American Journal of Orthodontics and Dentofacial Orthopaedics*.

[8] Shiu-yin C., Chu V., Ki Y. (2012). A retrospective study on 69 cases of maxillary tooth transposition. *Journal of Oral Science*.

[9] Burnett S. E. (1999). Prevalence of maxillary canine-first premolar transposition in a composite African sample. *Angle Orthodontist*.

[10] Umweni A. A., Ojo M. A. (1997). The frequency of tooth transpositions in Nigerians, and its possible aetiologic factors and clinical implications. *Journal of the Dental Association of South Africa*.

[11] Sandham A., Harvie H. (1985). Ectopic eruption of the maxillary canine resulting in transposition with adjacent teeth. *Tandlaegebladet*.

[12] Thilander B., Jakobsson S. O. (1968). Local factors in impaction of maxillary canines. *Acta Odontologica Scandinavia*.

[13] Dyal P. K., Shodhan K. H., Dave C. J. (1983). Transposition of canine with traumatic etiology. *Journal of Indian Dental Association*.

[14] Plunket D. J., Dysart P. S., Kardos T. B., Herbison G. P. (1998). A study of transposed canines in a sample of orthodontic patients. *British Journal of Orthodontics*.

[15] Budai M., Ficzere I., Gábris K., Tarjan I. (2003). Frequency of transposition and its treatment at the department of orthodontics of Semmelweis University in the last five years. *Fogorvosi Szemle Journal*.

[16] Peck S., Peck L. (1995). Classification of maxillary tooth transpositions. *American Journal of Orthodontics and Dentofacial Orthopaedics*.

[17] Peck L., Peck S., Attia Y. (1993). Maxillary canine-first premolar transposition associated dental anomalies and genetic basis. *Angle Orthodontist*.

[18] Peck S., Peck L., Kataja M. (1998). Mandibular lateral incisor-canine transposition, concomitant dental anomalies, and genetic control. *Angle Orthodontist*.

[19] Ciarlantini R., Melsen B. (2007). Maxillary tooth transposition: correct or accept?. *American Journal of Orthodontics and Dentofacial Orthopedics*.

[20] Weeks E. C., Power S. M. (1996). The presentations and management of transposed teeth. *British Dental Journal*.

[21] Feichtinger C., Rossiwall B., Wunderer H. (1977). Canine transposition as autosomal recessive trait in an inbred kindred. *Journal of Dental Restoration*.

[22] Papadopoulos M. A., Chatzoudi M., Kaklamanos E. G. (2010). Prevalence of tooth transposition. *Angle Orthodontist*.

[23] Venkataraghavan K., Anantharaj Athimuthu P. P., Jagadeesh R. B. (2014). Transposition of mandibular lateral incisor – canine (Mn I2.C) associated with hypodontia: a review and rare clinical case. *Journal of Clinical and Diagnostic Research*.

[24] Qamar C. R., Riaz M. (2010). Transposition of teeth: a review of the literature. *Pakistan Orthodontic Journal*.

[25] Demir A., Basciftci F. A., Gelgör I. E., Karaman A. I. (2002). Maxillary canine transposition. *Journal of Clinical Orthodontics*.

[26] Ruprecht A., Batniji S., El-Neweihi E. (1985). The incidence of transposition of teeth in dental patients. *Journal of Paedodontics*.

[27] Joshi M. R., Bhatt N. A. (1971). Canine transposition. *Oral Surgery, Oral Medicine and Oral Pathology*.

[28] Filho C., Cardoso M. A., An T. L., Bertoz F. A. (2007). Maxillary canine-first premolar transposition. *Angle Orthodontist*.

[29] Tukkahraman H., Sayın M., Yılmaz H. H. (2005). Maxillary canine transposition to incisor site: a rare condition. *Angle Orthodontist*.

[30] Ely N. J., Sherriff M., Cobourne M. T. (2006). Dental transposition as a disorder of genetic origin. *European Journal of Orthodontics*.

[31] Shapira Y. (1980). Transposition of canines. *Journal of American Dental Association*.

[32] Camilleri S. (2005). Maxillary canine anomalies and tooth agenesis. *European Journal of Orthodontics*.

[33] Yilmaz H. H. (2005). Prevalence of tooth transpositions and associated dental anomalies in Turkish population. *Dento-maxillofacial Radiology*.

[34] Cannavale R., Matarese G., Isola G., Grassia V., Perillo L. (2013). Early treatment of an ectopic premolar transposition. *American Journal of Orthodontics and Dentofacial Orthopedics*.

[35] Oesterle L. J., Cronin R. J., Ranly D. M. (1993). Maxillary implants and the growing patient. *International Journal of Maxillofacial Implantology*.

[36] Nambiar S., Mogra S., Shetty S. (2014). Transposition of teeth: a forensic perspective. *Journal of Forensic Dental Sciences*.

[37] Shapira Y., Kuftinec M. M. (1989). Tooth transpositions- a review of the literature and treatment considerations. *Angle Orthodontist*.

[38] Rabie A. B., Wong R. W. (1999). Bilateral transposition of maxillary canines to the incisor region. *Journal of Clinical Orthodontics*.

[39] Kurodas S., Kurodab Y. (2005). Nonextraction treatment of upper canine-premolar transposition in an adult patient. *Angle Orthodontist*.

[40] Shapira Y., Kuftinec M. M. (2001). A unique treatment approach for maxillary canine-lateral incisor transposition. *American Journal of Orthodontics and Dentofacial Orthopedics*.

[41] Sarver D. M., Ackerman M. B. (2003). Dynamic smile visualization and quantification: part 1. Evolution of the concept and dynamic records for smile capture. *American Journal of Orthodontics and Dentofacial Orthopedics*.

[42] Sarver D. M., Ackerman M. B. (2003). Dynamic smile visualization and quantification: part 2. Smile analysis and treatment strategies. *American Journal of Orthodontics and Dentofacial Orthopedics*.

[43] Luchessi W., Nortjé C. J. (1988). Suitability of the panoramic radiograph for assessment of mesiodistal angulation of teeth in the buccal segments of the mandible. *American Journal of Orthodontics*.

[44] Motloba D. P., Sethusa M. P. S., Ayo-Yusuf O. A. (2002). The psychological impact of malocclusion on patients seeking orthodontic treatment at a South African oral health training centre. *South African Dental Journal*.

[45] Nanda R. (2005). Dr. Ravindra Nanda on his treatment philosophy part - I. *Journal of the Indian Orthodontic Society*.

